# Age, season and spatio-temporal factors affecting the prevalence of *Echinococcus multilocularis *and *Taenia taeniaeformis *in *Arvicola terrestris*

**DOI:** 10.1186/1756-3305-4-6

**Published:** 2011-01-19

**Authors:** Pierre Burlet, Peter Deplazes, Daniel Hegglin

**Affiliations:** 1 Institute of Parasitology, University of Zurich, Winterthurerstr. 266a, CH-8057 Zurich, Switzerland

## Abstract

**Background:**

*Taenia taeniaeformis *and the related zoonotic cestode *Echinococcus multilocularis *both infect the water vole *Arvicola terrestris*. We investigated the effect of age, spatio-temporal and season-related factors on the prevalence of these parasites in their shared intermediate host. The absolute age of the voles was calculated based on their eye lens weights, and we included the mean day temperature and mean precipitation experienced by each individual as independent factors.

**Results:**

Overall prevalences of *E. multilocularis *and *T. taeniaeformis *were 15.1% and 23.4%, respectively, in 856 *A. terrestris *trapped in the canton Zürich, Switzerland. Prevalences were lower in young (≤ 3 months: *E. multilocularis *7.6%, *T. taeniaeformis *17.9%) than in older animals (>7 months: 32.6% and 34.8%). Only 12 of 129 *E. multilocularis*-infected voles harboured protoscoleces. Similar proportions of animals with several strobilocerci were found in *T. taeniaeformis *infected voles of <5 months and ≥5 months of age (12.8% and 11.9%). Multivariate analyses revealed strong spatio-temporal variations in prevalences of *E. multilocularis*. In one trapping area, prevalences varied on an exceptional high level of 40.6-78.5% during the whole study period. Low temperatures significantly correlated with the infection rate whereas precipitation was of lower importance. Significant spatial variations in prevalences were also identified for *Taenia taeniaeformis*. Although the trapping period and the meteorological factors temperature and precipitation were included in the best models for explaining the infection risk, their effects were not significant for this parasite.

**Conclusions:**

Our results demonstrate that, besides temporal and spatial factors, low temperatures contribute to the risk of infection with *E. multilocularis*. This suggests that the enhanced survival of *E. multilocularis *eggs under cold weather conditions determines the level of infection pressure on the intermediate hosts and possibly also the infection risk for human alveolar echincoccosis (AE). Therefore, interventions against the zoonotic cestode *E. multilocularis *by deworming foxes may be most efficient if conducted just before and during winter.

## Background

Population dynamics of organisms in temperate zones are generally shaped by seasonal variations. Parasites living within their hosts are protected from the direct impact of season-related factors like temperature or humidity but they usually have free living stages that can directly be affected by adverse environmental conditions. The understanding of how meteorological factors and seasonal changes affect the population dynamics of zoonotic parasites can contribute to better understand their epidemiology and to develop efficient control strategies.

In many parts of Europe, the zoonotic fox tapeworm *Echinococcus multilocularis *has benefited from increasing fox (*Vulpes vulpes*) populations and the invasion of foxes into urbanized areas during the last two decades [1-4]. In many cities of Switzerland, Germany and France, the life cycle of *E. multilocularis *is established in urban settings [2,5-7]. As a consequence, the incidence of human alveolar echinococcosis (AE) has increased in Switzerland by a factor of 2.6 during the first five years of the 21^st ^century as compared with the preceding five year period [8]. Human AE is an expensive disease to manage [9] and the frequently life-long treatment is very demanding for the affected patients. Therefore, there is a need to better understand the factors which affect the transmission dynamics of this parasite.

In experimental studies, it has been shown that the eggs of *E. multilocularis *can survive several months in a cold and humid environment, which is typical for winters in central Europe, but only a few days when exposed to dry and hot conditions prevailing in summers [10]. It therefore could be expected that *E. multilocularis *eggs excreted by foxes can accumulate during winter resulting in a higher infection pressure during this period compared to the rest of the year.

In Europe, the natural life cycle of *E. multilocularis *depends on the predator-prey relationship between foxes as the most important definitive hosts and *Arvicolidae *(voles), mainly the species *Microtus arvalis *and *Arvicola terrestris*, as intermediate hosts [11]. *Arvicola terrestris *and *M. arvalis *have a short life expectancy ranging from several months to rarely over 1 year [12]. Their population densities and structures are strongly affected by perennial cycles [13,14] and seasonal changes. Population densities of voles are generally highest in autumn and lowest in spring when the age structure of populations is strongly shaped by a higher proportion of old animals due to reduced reproduction during winter [15-17]. To understand the seasonal variation in the epidemiology of *E. *multilocularis, it is therefore important to know to what extent different age classes of the intermediate hosts develop the infective stages (protoscoleces) for the final hosts.

We investigated the influence of temporal and spatial factors on the prevalence of *E. multilocularis *in *A. terrestris*, the most abundant intermediate host in the city of Zürich, Switzerland. Furthermore, we analysed how age affects the prevalence and the development of protoscoleces and whether low temperatures (as a proxy for the winter season) and high humidity correlate with infection risk. The same analyses were undertaken for *Taenia taeniaeformis*, another *taeniid *species with domestic cats as principal definitive host and *A. terrestris *as a frequent intermediate host. Eggs of *Taenia *species have a similar resistance to freezing [18] and desiccation [19] as those of *E. multilocularis*, suggesting similarities concerning seasonal variations in the infection pressure on intermediate hosts.

## Materials and methods

### Study area and animals

**Figure 1:**
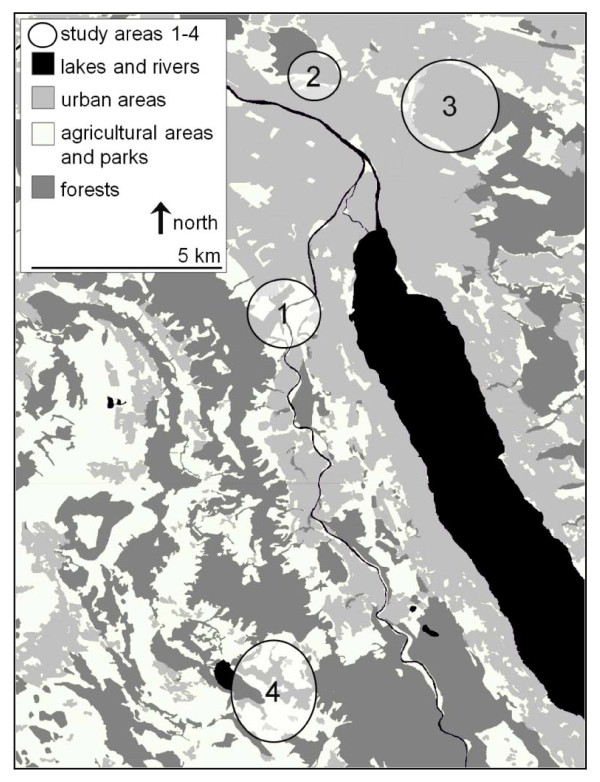
**Study areas in the canton of Zurich, Switzerland**. Areas 1-3 are situated along the urban periphery of the city of Zurich, area 4 is located in the the municipality of Rifferswil. Number of investigated water voles (*Arvicola terrestris*): N = 99 (area 1), N = 28 (area 2), N = 125 (area 3), N = 604 (area 4).

The study was conducted in the periphery of the city of Zurich, Switzerland, and in the nearby municipality of Rifferswil (Figure 1). From March 2007 to June 2008, a total of 856 *A. terrestris*, 252 animals in Zurich and 604 in Rifferswil, were collected. These animals were not trapped for the purpose of this study but rather in the framework of a continuous control program to avoid agricultural damages on grassland areas. Field workers used unbaited Topcat traps (Topcat GmBH, L'Auberson; Switzerland) and tongue traps (Hauptner Instrumente GmbH, Dietlikon, Switzerland), which were placed in vole galleries.

The voles were either dissected immediately after trapping or stored in a chest freezer at a constant temperature of -20°C prior to dissection. Careful examination was performed at the opening of thoracic and peritoneal cavities, and organs, in particular the liver, were attentively examined for lesions. Metacestodes were collected and identified after morphological characteristics. *Taenia taeniaeformis *was determined by counting all lucent, round-shaped vesicles of 3-10 mm size. All other lesions with a diameter of > 3 mm were cut into small pieces and investigated for the presence of protoscoleces of *E. multilocularis*. If protoscoleces were present, the metacestode material was squashed in a sieve with 1 mm mesh size and washed with PBS. Protoscoleces were counted under a binocular microscope in a petri dish. If more than 100 protoscoleces were present, 3 diluted subsamples of 100 μl were counted microscopically and the total number was calculated. Visually unidentifiable lesions were further investigated after proteinase K digestion by a PCR specific for *E. multilocularis *[20].

#### Age determination of rodents

The absolute age was calculated by measuring the weight of dry crystalline lenses [21,22] according to Burlet et al. [23]. In short, after dissection, eyes were put directly into formalin (10%) for fixation over a period of 4 weeks. Lenses were then removed from the eye, air-dried at +80°C over a period of 48 hours and immediately weighted. The age of individual voles was calculated by the formula ${x=e}^{\frac{y-1.858}{1.202}}$, where *x *is the age in months and *y *the eye lens weight in mg. As freezing increases lens weights of *A. terrestris *by 3.3%, lens weights of frozen lenses were divided by 1.033 to obtain a correct age estimate [23].

#### Determination of seasonal and climatic factors

The date of birth of each vole was calculated based on its age and trapping day. To analyse the influence of meteorological factors, the means of day temperature (measured 5 cm above ground) and of precipitation experienced by each individual was calculated (data source: Swiss Meteorological Institute MeteoSwiss; weather station Zürich Fluntern, 8°34'/47°23').

### Statistical analyses

Prevalences of *E. multilocularis *and *T. taeniaeformis *were analysed using logistic regression models. The variables age (age of individuals expressed in months), period (time when an individual was trapped: March - June 2007, July - October 2007, November 2007 - February 2008, March - June 2008), area (trapping areas 1-4, Figure 1), mean day temperature [°C] and mean precipitation per day of living [mm] were selected as independent variables for the modelling procedure.

Models were fitted using all possible combinations of the selected predictor variables. Best models were selected using Akaike's information criterion (AIC, [24]) corrected for small samples sizes. Only models with ΔAICc < 2 compared to the model with the lowest AICc were selected. Akaike model weights were calculated [25] to determine the degree by which a model was supported by the data.

Logistic regressions were calculated using SPSS 17.0 [24]. Maximum likelihood estimate of k was used to calculate the degree of overdispersion of the number of protoscoleces in infected rodents. This parameter of negative binomial distribution tends towards 0 with increasing accumulation of parasites [26].

## Results

**Figure 2:**
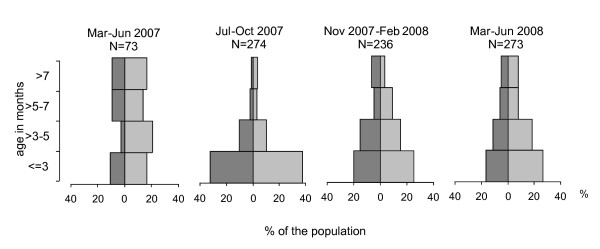
**Age pyramids of *Arvicola terrestris *in the canton of Zurich, Switzerland, in different seasons**. The single pyramid segments represent the percentage of the population trapped during the corresponding period. Dark grey: males, light grey: females.

Age determination revealed strong shifts in the age structure of the *A. terrestris *population over time (Figure 2). During the first period (March-June 2007) the portion of animals older than 5 months (48.6%, CI 95% 36.9%-60.6%) was significantly higher than in the same period one year later (March-June 2008: 26.8%, 21.7%-32.5%). Mean day temperatures per month during the second year of the study (July 07 - June 08) were consistently (unless August 2007 and May 2008) higher than during the preceding year (in average 2.0°C).

**Figure 3:**
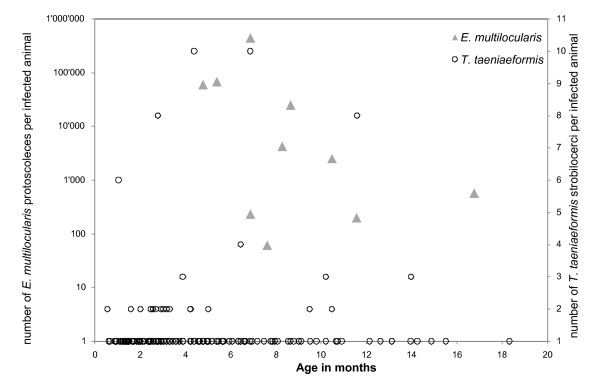
**Numbers of *Echinococcus multilocularis *protoscoleces and *Taenia taeniaeformis *strobilocerci in trapped *Arvicola terrestris***.

Liver lesions were observed in 270 of 856 dissected *A. terrestris *(31.5%, CI 95% 28.4%-34.8%). The overall prevalence rate of *E. multilocularis *was 15.1% (12.7%-17.6%), and protoscoleces were found in 12 animals corresponding to 1.4% (0.7%-2.4%) of all studied animals or 9.3% (4.9%-15.7%) of the *E. multilocularis*-positive animals. Animals older than 7 months were more than 4-times more frequently infected than animals ≤ 3 months. Furthermore, none of the animals under 3 months of age harboured protoscoleces (Table 1), and the youngest animal with protoscoleces was 3.2 months of age. In 10 animals the protoscolex burden was determined. The maximum likelihood estimate of k = 0.16 indicates a heavily overdispersed protoscolex burden. Four animals (40%) harboured between 61 and 568 protoscoleces (together 1057 protoscoleces), representing 0.2% of the total number, five animals had between 2492 and 67'550 protoscoleces, and the extrapolated number of protoscoleces was 451'540 in one animal, representing 73.8% of this parasite stage identified in this study. However, no relation between age and the number of protoscoleces was identified (Spearman R = -0.52, p = 0.12; Figure 3).

**Table 1:** Prevalence rates of taeniid infections in trapped *Arvicola terrestris *of different age classes

age class in months (N animals)	≤ 3 (N = 436)		>3-5 (N = 227)		>5-7 (N = 101)		>7 (N = 92)	
*E. multilocularis*	7.6	(5.3-10.5)	19.4	(14.5-25.1)	21.8	(14.2-31.1)	32.6	(23.2-43.2)
*E. multilocularis *protoscoleces	0.0	(0.0-0.7)	1.3	(0.3-3.8)	3.0	(0.6-8.4)	6.5	(2.4-13.7)
*T. taeniaeformis*	17.9	(14.4-21.8)	24.2	(18.8-30.3)	34.7	(25.5-44.8)	34.8	(25.1-45.4)
*T. crassiceps*	0.9	(0.3-2.3)	2.6	(1.0-5.7)	5.0	(1.6-11.2)	0.0	(0.0-3.2)

95% confidence intervals are shown in brackets (N total = 856).

The overall prevalence of *T. taeniaeformis *was 23.4% (20.6%-26.3%). Animals older than 5 months were roughly 2-times more frequently infected than animals ≤ 3 months (Table 1). Most infected animals harboured one *T. taeniaeformis *strobilocercus but 25 out of 200 infected animals had 2-10 strobilocerci (Figure 3). The proportion of multiple infections was not dependent on the age of the animals (Spearman R = 0.02, p = 0.82): Seventeen of 133 (12.8%) infected animals of <5 months of age and 8 of 67 (11.9%) infected animals of ≥ 5 months of age had more than one strobilocercus. In total, five of the ten animals with known *E. multilocularis *protoscoleces burden were simultaneously infected with *T. taeniaeformis*, which is a significant higher proportion than expected by chance (Actus randomization test, p<0.05). Three of these five animals harboured 2, 8 and 10 strobilocerci and, interestingly, the animal with 10 strobilocerci also had the highest protoscoleces burden. In addition, 1.9% (1.1%-3.0%) of all animals were infected with *T. crassiceps*. Scoleces of this species were mostly found in subcutaneous cysts but also pleural cavities.

**Figure 4:**
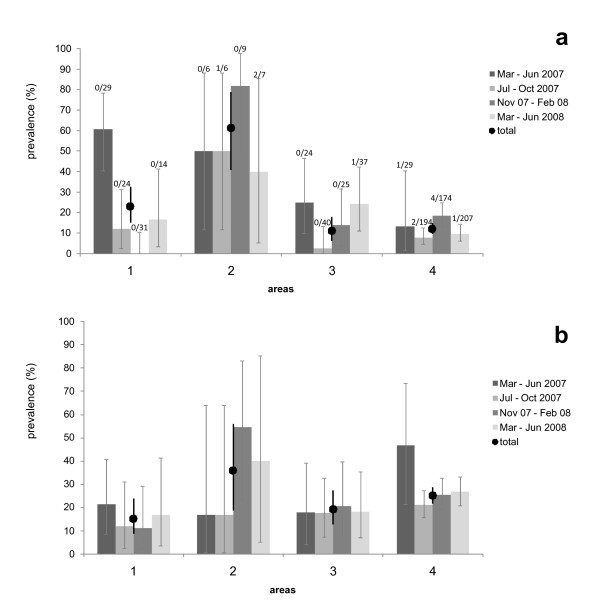
**Temporal prevalences of *Echinococcus multilocularis *and *Taenia taeniaeformis *strobilocerci in 4 study areas**. a) Prevalences and 95% confidence intervals of *Echinococcus multilocularis *(undifferentiated and protoscoleces-containing metacestodes), b) prevalences and 95% confidence intervals of *Taenia taeniaeformis *strobilocerci. Overall prevalence rates per area are symbolised by circles. For *E. multilocularis*, the number of voles with protoscoleces and the total number of studied individuals are given above the associated bars (N total = 856 *Arvicola terrestris*). Study areas are shown in Figure 1.

The model selection procedure for *E. multilocularis *infections revealed two best models (ΔAICc < 2; Table 2), containing 'age', 'period', 'area', 'mean day temperature' and 'mean precipitation' as factors for explaining the prevalence (Table 3). Prevalence rates of *E. multilocularis *differed strongly between the four trapping areas (Figure 4a) and ranged between 11.2% (95% CI 12.7-27.2, area 3) and 60.7% (CI 40.6-78.5%, area 2). Furthermore, low temperatures significantly increased the infection risk (Table 3). The second best model suggests that higher precipitation is associated with a higher infection risk but this effect was not significant (Table 3).

**Table 2:** Factors affecting prevalences of *Echinococcus multilocularis *and *Taenia taeniaeformis*

factors included in best models	AICc	ΔAICc	AICc weight
a)E. multilocularis*:			
age, period, area, mean day temperature	-244.04	0	0.57
age, period, area, mean day temperature, mean precipitation	-243.45	0.59	0.43

b)T. taeniaeformis**:			
age, area	51.08	0.00	0.42
age, area, mean day temperature	52.22	1.14	0.24
age, area, period, mean day temperature	52.75	1.67	0.18
age, area, mean precipitation	52.94	1.87	0.16

* Null model AICc = -131.22

** Null model AICc = 83.73

All factors are shown that were included in the best models (ΔAICc<2, N = 856).

**Table 3:** Odds ratios of factors explaining prevalences of *Echinococcus multilocularis *and *Taenia taeniaeformis *in *Arvicola terrestris*

	*E. multilocularis*				*T. taeniaeformis *strobilocerci							
	
	best model		2^nd ^best model		best model		2^nd ^best model		3^rd ^best model		4^th ^best model	
	
Model factors	OR	95% CI	OR	95% CI	OR	95% CI	OR	95% CI	OR	95% CI	OR	95% CI
Age	1.13	1.06-1.20	1.13	1.06-1.20	1.13	1.08-1.20	1.13	1.07-1.20	1.13	1.07-1.20	1.13	1.07-1.20
Period												
*-Mar-Jun 07 vs Mar-Jun08*	3.92	1.96-7.81	3.58	1.83-6.98					0.88	0.47-1.65		
*-Jul-Oct 07 vs Mar-Jun08*	1.70	0.75-3.89	1.70	0.75-3.83					0.95	0.63-1.45		
*-Nov07-Feb08 vs Mar-Jun08*	1.83	1.07-3.14	1.70	1.01-2.85					1.05	0.69-1.58		
Area												
*-Area 1 vs 4*	1.54	0.85-2.80	1.55	0.86-2.81	0.51	0.28-0.92	0.51	0.28-0.91	0.53	0.29-0.71	0.51	0.28-0.91
*-Area 2 vs 4*	8.60	3.60-20.50	8.63	3.61-20.66	1.05	0.45-2.46	1.09	0.46-2.55	1.07	0.45-2.51	1.06	0.45-2.48
*-Area 3 vs 4*	0.94	0.49-1.81	0.94	0.48-1.80	0.47	0.27-0.80	0.49	0.29-0.86	0.48	0.28-0.82	0.47	0.28-0.82
Mean day temperature	0.90	0.84-0.96	0.92	0.87-0.97			0.99	0.96-1.02				
Mean precipitation			1.13	0.90-1.43							0.98	0.88-1.09
Constant	0.09	-	0.11	-	0.22	-	0.26	-	0.22	-	0.24	-

Shown are all odds ratios (OR) and 95% confidence intervals (95%CI) for the factors of the best models (ΔAICc < 2, N = 856).

The model selection procedure for *T. taeniaeformis *revealed 4 best models (ΔAICc < 2; Table 2) which include the factors 'age', 'period', 'area', 'mean day temperature' and 'mean precipitation' as factors explaining parasite prevalence (Table 3). In animals with ages of 5 months or higher, the prevalence was significantly higher than in juvenile animals (Table 1), and prevalences were lower in area 1 than in area 4 (Table 3 and Figure 4b). Although each of the three factors 'period', 'mean temperature' and 'mean precipitation' entered one of the four best models, the 95% confidence intervals of the odds ratios strongly overlapped the value 1 for all three factors.

## Discussion

### Prevalence of *E. multilocularis*

This study shows that the transmission dynamics of the two taeniid species *E. multilocularis *and *T. taeniaeformis *are significantly affected by spatial factors. The prevalence rates of *E. multilocularis *in *A. terrestris *were significantly higher (95% CI: 40.6-78.5%) in one study area as compared to the others and surpassed, to our knowledge, the highest ever reported prevalence of 39% in intermediate hosts in Central Europe [27]. This finding confirms the occurrence of micro-foci [28-30] with exceptional high *E. multilocularis *infection pressure in densely populated areas and possibly reflects the high fox densities in urban and periurban areas [2,3,31].

Transmission dynamics of the two investigated taeniid species can be affected by various host-related factors. In addition to the densities of final (foxes for *E. multilocularis *and domestic cats for *T. taeniaeformis*) and intermediate hosts [32-34], the predation activity of final hosts [17,35] can play an important role in transmission [7,36]. Furthermore, temporal fluctuations of prevalences can origin from shifts in the age structure of populations [37]. Once a metacestode has established, it can be detected for the rest of the rodent's life. Therefore, infections accumulate with increasing age in single vole generations and prevalences increase. In previous studies done in Zurich and the city of Geneva, *E. multilocularis *prevalences were 10.7 and 9.2%, in adult voles respectively, and 1.3% in subadults and juveniles [20,38]. In this study, we also recorded a higher prevalence in adult voles. Furthermore, we documented an increase of prevalence rates over several age classes (Table 1).

The age structure of voles is closely related to seasonal factors. In early spring, old animals predominate as reproduction is low in winter [16,17,37]. Nevertheless, season-related age structure can vary considerably from year to year, as shown by our data with a higher proportion of old overwintering voles in spring 2007 (Figure 2) after an extraordinary mild winter [39,40]. As age strongly affects parasite prevalence, such temporal variations in the age structure over years can hamper the detection of seasonal variation in the infection pressure.

As shown in this study, the determination of the absolute age of intermediate hosts can help to overcome such methodological limitations. Based on this data, it was possible to calculate for each individual rodent to which temperatures it was exposed during its live. Hence, it could be demonstrated that low day temperatures typical for the winter season correlate with a higher infection rate. Absolute age estimates were also considered in the only study we are aware of that also described a clear season-related infection pattern of *E. multilocularis *[37]. As in our study for *A. terrestris*, seasonal patterns of infection were found for *M. arvalis *with highest prevalences of *E. multilocularis *in spring in old over-wintered animals which had acquired their infections in winter (from October to April).

Staubach et al. [41] demonstrated that *E. multilocularis*-infected foxes are more frequently found in areas with high soil moisture. Correspondingly, our second best model explaining the prevalence of *E. multilocularis *in *A. terrestris *included the factor precipitation. However, the effect size of this factor alone was too small to demonstrate a clear relationship with the infection risk. Soil moisture is not only affected by precipitation but also by many other factors (e.g. temperature, vegetation growth and sun exposition [Hagan 1955]) and a direct measure of soil moisture could be a better predictor than the amount of precipitation to explain the infection pressure.

The fact that living during periods with low temperatures contributed significantly to the infection risk with *E. multilocularis *underlines the epidemiological relevance of the experimental study of Veit and colleagues [10] showing that *E. multilocularis *eggs survive for months under cold conditions but die within a few hot and dry days. Another aspect that possibly contributes to seasonal patterns in the parasite's transmission dynamics is a seasonal pattern of the predation on *A. terrestris *and other rodents by foxes [7,17,36]. These studies revealed higher predation rates in autumn with a larger availability of voles than in spring, when rodent density are generally considerably lower [7,42]. The higher consumption possibly explains higher prevalence rates in foxes during winter months as revealed by several studies in high endemic areas [43,44] and consequently, a higher contamination of the environment with *E. multilocularis *eggs.

The presence of protoscoleces was clearly related to the age of the investigated animals. The low number of animals with protoscoleces made it impossible to perform multivariable analyses in order to investigate seasonal effects on the prevalence of protoscoleces harbouring animals. However, it is expected that season-related changes in the age structure of the intermediate host population cause that there is a higher proportion of animals harbouring protoscoleces during winter and early spring, before the start of reproduction. This does not necessarily mean that foxes are exposed to a higher infection pressure during this period, as absolute density of voles and predation on rodents by foxes is considerably lower during spring and early summer [17,36,45].

### Prevalences of *T. taeniaeformis*

Similar to *E. multilocularis*, *T. taeniaeformis *causes lifelong infections in intermediate hosts and is more prevalent in older animals [20,38,46-48]. Furthermore, eggs of different taeniid species have a similar resistance to environmental conditions as those of *E. multilocularis *[10,19]. Accordingly, two other studies reported higher prevalence rates in overwintered intermediate hosts [15,47]. In contrast, we found no clear association of low temperatures and high precipitation with higher prevalence rates of *T. taeniaeformis*. The eggs of this parasite might be less sensitive to hot and dry weather condition due to the special defecation behavior of domestic cats which usually burry their droppings into loose soil where taeniid eggs are less exposed to adverse weather conditions. Further, the loose ground of the burrows of *A. terrestris *could be a good substrate for cats to defecate.

Contrary to Reperant and colleagues [38], we found significant spatial variations in the prevalence of *T. taeniaeformis*. However, in contrast to Le Pesteur [15], the observed pattern did not correspond to the spatial variation of *E. multilocularis *prevalence rates. Probably, these different spatial patterns are caused by different distributions of fox and cat densities. Interestingly, the lowest prevalence rates for *T. taeniaeformis *were found in an area (area 1) where many people walk their dogs and this might contribute to a lower presence of domestic cats.

Although 23% of all investigated animals were infected with *T. taeniaeformis*, only 13% of these 200 animals harbored more than one strobilocercus. Furthermore, increasing age was not linked to a higher amount of animals with more than one strobilocercus and the prevalences were similar in animals >7 months and those of 5 to ≤ 7 months (Table 1). This parasite has not a proliferative growth like *E. multilocularis *[49] and it is unlikely that it caused an increase of the mortality of infected intermediate hosts which could explain such an asymptotic increase of prevalence along the age classes. Possibly *T. taeniaeformis *was hyperendemic in *A. terrestris *and regulated by a concomitant immunity. Such regulations have been demonstrated with experimental *T. taeniaeformis *infections of rats [50,51] and by epidemiological investigations of sheep and goats infected with *T. hydatigena *[52]. Interestingly, the fact that heavily *E. multilocularis*-infected voles had more *T. taeniaeformis *strobilocerci than expected indicates an immuno-suppression driven by *E. multilocularis *metacestode [53], which counter-acts the protective immune mechanisms against super-infections with *T. taeniaeformis*.

## Conclusions

Our results demonstrate that the availability of absolute age estimates of intermediate hosts can be crucial to detect season-related variations in the infection pressure by taeniid species. As shown for *E. multilocularis*, infection pressure on voles can vary considerably within a small spatial scale and along different seasons. This knowledge possibly can also contribute to model spatial and temporal variation of the infection risk for human. Based on the presented results, we suggest that reducing the infection pressure on intermediate host and presumably as well on humans by the delivery of anthelmintic baits for foxes is more effective during the cold and humid winter season than during the rest of the year.

## Competing interests

The authors declare that they have no competing interests.

## Authors' contributions

PB did the laboratory work, analyzed the data, participated in the statistical analyses and drafted the manuscript. PD and DH designed and supervised the study and critically revised the manuscript. Additionally, DH contributed to data analyses and statistics. All authors read and approved the final manuscript.
